# Physicochemical and Microstructural Properties of Polymerized Whey Protein Encapsulated 3,3′-Diindolylmethane Nanoparticles

**DOI:** 10.3390/molecules24040702

**Published:** 2019-02-15

**Authors:** Abbas Khan, Cuina Wang, Xiaomeng Sun, Adam Killpartrick, Mingruo Guo

**Affiliations:** 1 Department of Food Science, College of Food Science and Engineering, Jilin University, Changchun 130062, China; abbaskhan9916@mails.jlu.edu.cn (A.K.); wangcuina@jlu.edu.cn (C.W.); sunxm15@mails.jlu.edu.cn (X.S.); 2 Food Science Corporation, Inc., Williston, VT 05495, USA; akillpartrick@foodsciencecorp.com; 3 College of Agriculture and Life Sciences, The University of Vermont, Burlington, VT 05405, USA; 4 Department of Food Science, Northeast Agriculture University, Harbin 150030, China

**Keywords:** 3,3′-diindolylmethane, polymerized whey protein, physicochemical properties, nanoparticles

## Abstract

The fat-soluble antioxidant 3,3′-diindolylmethane (DIM), is a natural phytochemical found in *Brassica* vegetables, such as cabbage, broccoli, and Brussels sprouts. The stability of this compound is a major challenge for its applications. Polymerized whey protein (PWP)-based DIM nanoparticles were prepared at different mass ratios of protein and DIM by mixing PWP and DIM followed by ultrasound treatment for 4 min. All the nanoparticles were studied for particle size, zeta potential, rheological and microstructural properties, and storage stability. The mean particle size of the PWP-based nanoparticles was significantly increased (*p* < 0.05) by the addition of DIM at different mass ratios, ranging from 241.33 ± 14.82 to 270.57 ± 15.28 nm. Zeta potential values of all nanoparticles were highly negative (greater than ±30 mV), suggesting a stable solution due its electrostatic repulsive forces. All samples exhibited shear thinning behavior (*n* < 1), fitted with Sisko model (R^2^ > 0.997). Fourier Transform Infrared (FTIR)spectra revealed that the secondary structure was changed and the absorption intensity for hydrogen bonding got stronger by further incorporating DIM into PWP. Transmission electronic microscopy (TEM) images showed spherical and smooth surface shape of the PWP-based nanoparticles. DIM encapsulated by PWP showed enhanced stability at 4, 37 and 55 °C for 15 days evidenced by changes in mean particle size and color (a*-value and b*-value) compared with control (DIM only). In conclusion, the polymerized whey protein based 3,3′-diindolylmethane nanoparticles are stable and the encapsulation may protect the core material from oxidation.

## 1. Introduction

3,3′-Diindolylmethane (DIM) is a natural phytochemical, the most important product of dietary indole-3-carbinol (I3C), and it can be found in all cruciferous vegetables. It is formed by acid-hydrolysis of glucobrassic in kale and *Brassica* species such as Brussels sprouts and broccoli [1]. DIM has a wide array of pharmacological activities and has shown inhibitory effects on oncogenesis, inflammation, progression, and tumor growth, which suggests its usefulness as a chemo preventive and in therapeutic functions [2,3]. DIM has potential ability to prevent different types of cancer [4,5], especially human breast cancer [6]. DIM has a promising effect to treat thyroid proliferative disease by promoting healthier estrogen metabolism [7] and can be used as an antibacterial [8]. However, the stability of DIM is a major challenge for its practical applications. In addition, the bioavailability of 3,3′-diindolylmethane (DIM) is poor due to its low solubility/high lipophilicity and limited oral absorption [9].

In order to increase the physicochemical stability and bioavailability of DIM, zein/carboxymethyl chitosan nanoparticles were produced to encapsulate 3,3-diindolylmethane to provide its controlled release for pharmaceutical/food applications [10]. Encapsulation could enhance the bioavailability, and improve the stability under harsh environmental conditions. Nanoparticles provide protection to the encapsulated compounds against photo and thermo-degradation [11]. In order to lengthen shelf life, preserve the functionality, offer controlled release, and improve chemical stability of different natural bioactive compound and drugs such as ascorbic acid, caffeic acid, β-carotene, anthocyanins, many biodegradable polymeric nanoparticles and delivery systems have been used [12,13,14]. Microencapsulation is a versatile and distinctive technique used in food, pharmaceutical, agricultural, and other fields by providing controlled environment and protect a lot of active substances [11,15,16,17]. Microencapsulation can control the interactions between the external and internal part by creating a microenvironment within the capsules, which protect sun stable food components against harsh environments, nutritional loss, maintains fragrance and offers controlled-release. Moreover, sensitive, reactive, and unstable additives can be protected through microencapsulation [10,18,19]. Microencapsulation is appropriate for biopharmaceutics and biomedicines, and food industry applications, particularly for high-value nutraceuticals and aliments. Previous studies have reported that microencapsulated DIM can increase oral bioavailability [20]

Whey protein is a by-product of cheese making, with major constituents of α-lactalbumin, and β-lactoglobulin, functional food processing ingredients. They possess some desirable functional properties in addition to nutritional quality [21]. Whey proteins can entrap hydrophobic compounds such as vitamin D_3_ [22]. Whey protein concentrates have been used as a wall material for encapsulation, due to their surface-active properties, and can form a protective coat around the core materials as a result of hydrophobic interactions [23,24]. However, limited studies have been reported regarding microencapsulation with polymerized whey protein as a wall material. It can be developed by heat treatment which can change the structure of proteins without a chemical cross-linking agent [25,26]. Moreover, whey proteins when heated above 70 °C can form gels or aggregates [27]. Heat treatment causes changes in the structure of proteins, which can lead to protein denaturation and aggregation, and this treatment is now used as an innovative technology in the food industry to enhance the emulsifying and functional properties of whey proteins [28].

Therefore, there is great interest in determining whether encapsulation of DIM in polymerized whey protein nanoparticles can improve its physicochemical and rheological properties, and protect it from harsh environmental conditions. In this study, the potential of polymerized whey protein for encapsulation of DIM under ultrasound treatment was studied. The protective effects on the stability of DIM were also studied.

## 2. Results and Discussion

### 2.1. ParticleSize and Zeta Potential of PWP–DIMNanoparticles

DIM was added to polymerized whey protein (PWP) with different mass ratios to optimize a stable formulation. All the formulations used in the study were found to be stable for a long period of time, except PWP/DIM with mass ratio 3:1, which was excluded from further analyses due to observed sedimentation (which started after 1day of storage). Particle size and zeta potential are important indicators of the formation of stable nanoparticles for medical and nutritional applications. The mean particle size, polydispersity index (PDI), and zeta potential of the samples are listed in Table 1. The mean particle size of PWP only (202.50 ± 1.56 nm) was significantly increased (*p* < 0.05) by the addition of DIM with different mass ratios ranging from 241.33 ± 14.82 to 270.57 ± 15.28 nm. This difference could be due to the entrapment of DIM in the whey protein nanoparticles by enhanced hydrophobic interactions, which results in an increased particle size [22]. Comparing the mean particle size of different mass ratios of PWP-based DIM nanoparticles, no significant difference was found, except for samples at 5:1 ratio with a mean particle size of 270.57 ± 15.28 nm, which was significantly higher (*p* < 0.05). It has been similarly reported that a significant increase in mean size with different core-to-wall ratio was seen by encapsulating ophylline in whey proteins [29]. Polymerized whey protein can successfully stabilize DIM for a long time. Previous research reported that WPC alone and in combination with Tween-80 were tried in the encapsulation of curcum into overcome its instability during processing [30]. 

The PDI value of all samples was less than 0.45, except for nanoparticles prepared with a higher mass ratio, i.e., 5:1, showing narrow distribution and good stability of the particle size [31]. A significant difference was observed for PDI values of the PWP alone and whey protein-based DIM nanoparticles (*p* < 0.05, Table 1). 

Zeta potential is a measure of the surface charge characteristics and stability against aggregation and coalescence [32,33]. The zeta potential values of polymerized whey protein nanoparticles with DIM and without DIM are shown in Table 1. The zeta potential value of PWP alone was (−30.26 ± 0.20 mV) and that of PWP-based DIM nanoparticles with different mass ratios i.e., 5:1, 10:1, 15:1, 20:1was −36.56 ± 0.37, −34.60 ± 1.12, −35.13 ± 0.83, and −35.40 ± 1.24 mV, respectively. In general, the absolute zeta potential values of all the samples were greater than ± 30 mV, suggesting that DIM-whey protein nanoparticles are more stable due to electrostatic repulsion [32]. Greater absolute values of zeta potential of nanodispersion could be considered indicative of physical stability due to more repulsive forces among the particles [34,35].

### 2.2. Rheological Properties of PWP–DIM Nanoparticles

**Figure 1:**
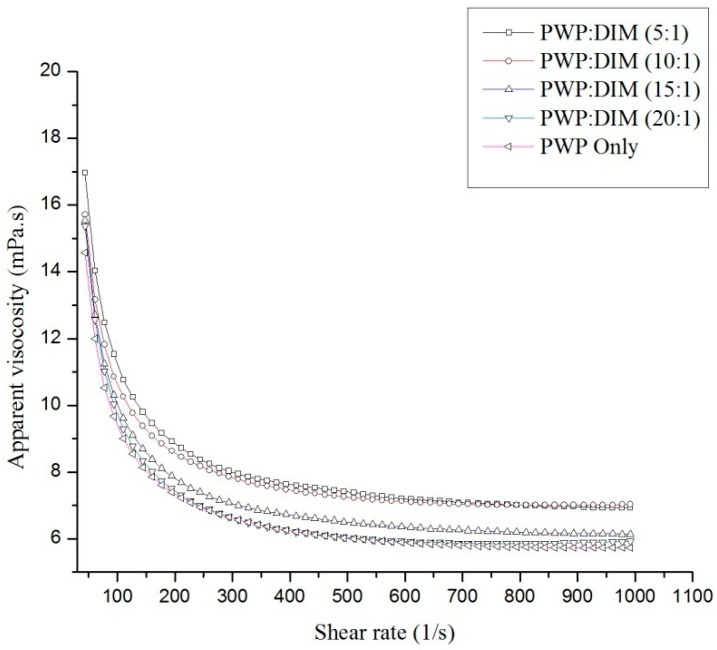
Behaviour of PWP and whey protein based DIM nanoparticles.

Figure 1 shows the flow curve of PWP alone and PWP/DIM with different mass ratios. All the samples with different ratios of DIM in PWP nanoparticles showed different curves, and it was clear that increasing the DIM mass in polymerized whey protein from 20:1 to 5:1 had a significant effect (*p* < 0.05) on the apparent viscosity (η) of the nanoparticles. Similar results for the dependence of nanoparticles’ apparent viscosity η_app_ on hydrocolloid concentration have been reported indifferent systems [36,37,38,39]. Moreover, the samples’ viscosity decreased with the increase in shear rate region and displayed an infinite shear viscosity (η_∞_) at the high shear rate. Based on the strong correlation (Table 2), a Sisko model [40] fitted the viscous flow behavior of PWPC/DIM nanoparticles well (R^2^ > 0.997 in all cases):

η_app_= *k*_0_ γ^n−1^ +η_∞_ (1)

where, η_app_ is the apparent viscosity, *k*_0_ is the consistency index, γ is the shear rate (s^−1^), η_∞_ is infinite shear rate viscosity, and *n* is the flow index.

The flow behavior of all the samples exhibited a shear-thinning Non-Newtonian or pseudo plastic (*n* < 1) behavior, and increased with increased of DIM content in the whey protein complexes, turning to a more Newtonian behavior (close to 1). In addition, by increasing the DIM content in PWP, the parameter η_∞_ is slightly increased, suggesting an increase in viscosity. The same rheology model (Sisko model) was used and the results showed a similar shear thinning flow behavior [36,41]. The results indicate that all the nanoparticles exhibit low viscosity and could be used in low viscosity foods.

### 2.3. FTIRSpectra of PWP–DIM Nanoparticles

**Figure 2:**
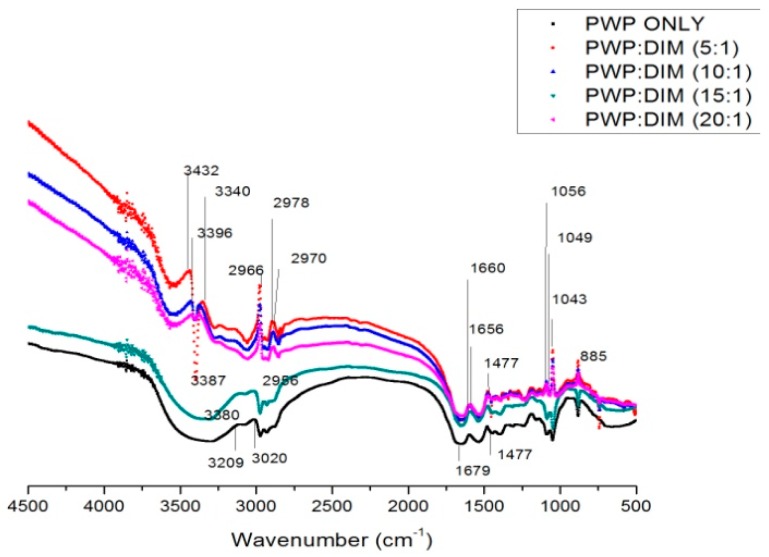
FT-IR spectra of PWP and PWP/DIM nanoparticles.

Figure 2 show the FTIR spectra of PWP and PWP-DIM nanoparticles with different ratios. In the FTIR spectra, a strong and interesting absorption band appeared in the range of 3200–3600 cm^−1^, representing hydrogen bonding. The hydrogen bond in PWP was only found at 3209 cm^−1^, and was shifted to 3432, 3387, 3396, 3380 and 3340 cm^−1^ in samples with PWP to DIM ratios of 5:1,10:1, 15:1 and 20:1, respectively, suggesting that hydrogen bonding might be formed between DIM and PWP [42]. In nanoparticles formation, hydrogen bonding is considered to be the major facilitating force [43]. On other hand, sharp peaks were confirmed at 2978, 2970, 2956, 2996 cm^−1^ in the spectra of PWP/DIM with different ratios, respectively, which revealed the C-H stretching of the aliphatic group (Figure 2) [44]. The peaks ranging from 1500–1700 cm^−1^ confirmed the presence of the C=O bond of the amide I band in the α-helical component of the β-lactoglobulin and α-lactalbumin secondary structures, which confirmed the presence of primary and secondary amine groups [45,46,47], and amide II band [48]. Moreover, the peak at 1040 to 1100 cm^−1^ in the spectra of DIM-loaded nanoparticles confirmed the N-H bonds and C-N stretching bond (amide III) [49]. It is speculated that hydrogen bonds play an important role by the addition of DIM into the protein complex. Similar results were found in previous studies on encapsulation of vitamin D_3_ with CMCS-soy protein complex nanoparticles [44]. From these results, it is concluded that maximum hydrogen bonding was gained by incorporating DIM into polymerized whey protein (PWP), which was found favorable for encapsulation.

### 2.4. Microstructure of PWP–DIM Nanoparticles

**Figure 3:**
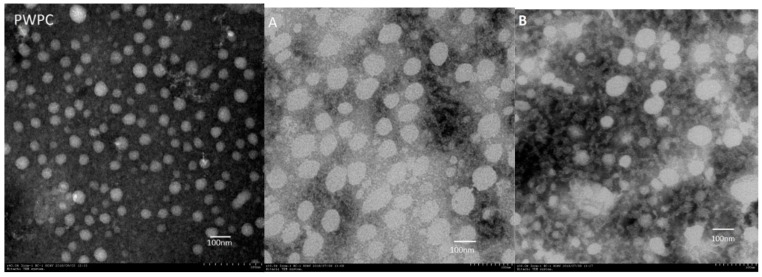

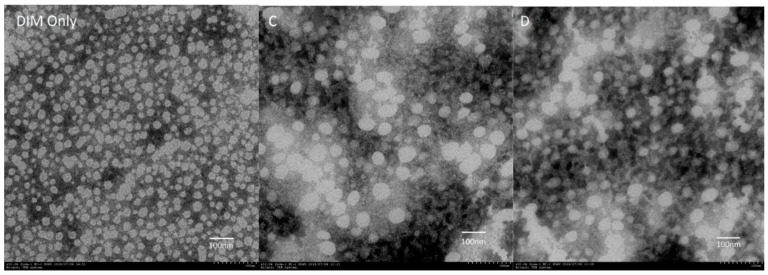
Images of DIM, PWPC only, and PWP/DIM with different mass ratios represented with (**A**) PWP/DIM (5:1), (**B**) PWP/DIM (10:1), (**C**) PWPC/DIM (15:1), and (**D**) PWP/DIM (20:1).

The morphological observations of DIM alone, PWP and PWP-DIM nanoparticles with different ratios are shown in Figure 3. The TEM image of DIM only showed an irregular and roughly spherical shape (Figure 3), and after encapsulation with polymerized whey protein (PWP) the shape of the particles became spherical and smooth. Moreover, the dispersion of DIM into polymerized whey protein (PWP) affected the size of the nanoparticles, and a significant change in the particle size was observed, justified by DLS data (Table 1). The newly obtained particles exhibited a spherical shape and smooth surface, which was in line with previous studies [19,50]. Another study also reported that upon encapsulating DIM with CMCS/zein, the nanoparticles exhibited smooth and spherical shapes [50]. When the DIM mass was increased from 20:1 to 5:1 in the polymerized whey protein (PWP) nanoparticles, the mean particle size was comparatively larger, which is consistent with the increase in particle size obtained in the DLS study. On the surface of the DIM, a cloud of whey protein can be seen, which seems to be the coating surface of polymerized whey protein.

### 2.5. Changes in Color and Absorbance of PWP–DIM nanoparticles during Storage

**Figure 4:**
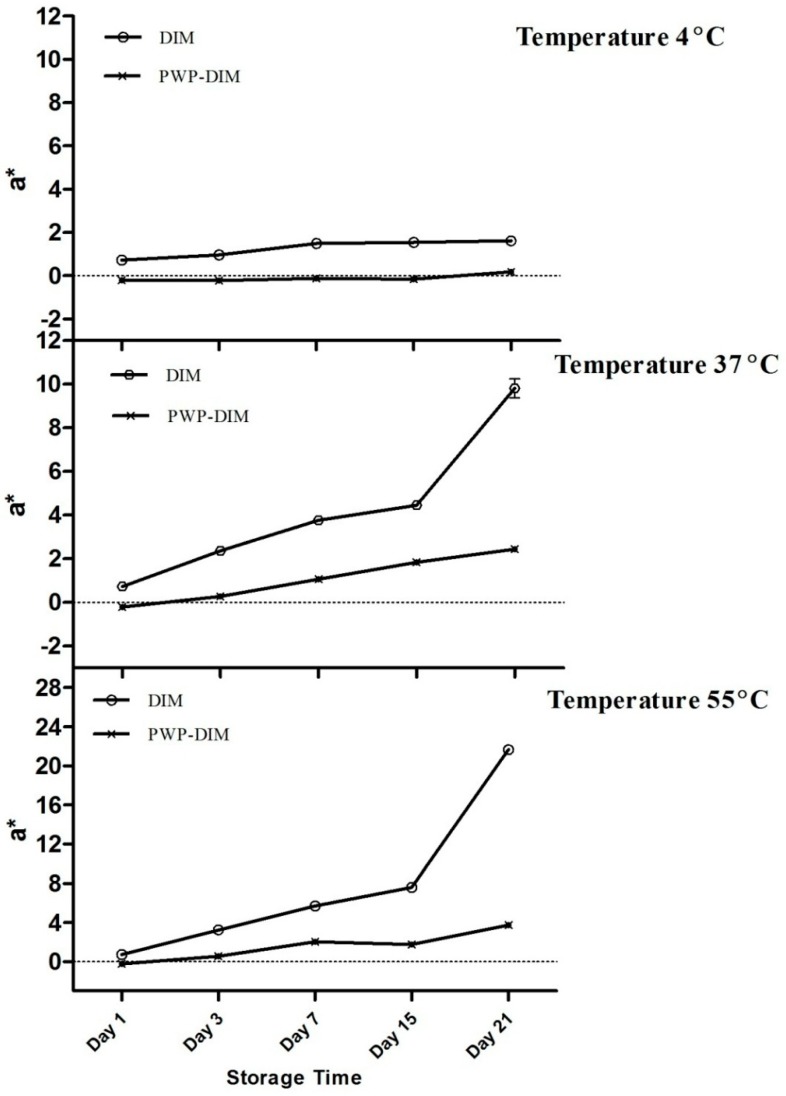
Effect of storage temperature (4, 37 and 55 °C) on the green-red axis (a*-value) of DIM and PWP/DIM with mass ratio (5:1) nanoparticles during storage. Values are mean ± SD (*n* = 3).

Changes in the color of control sample (pure DIM) and PWP/DIM nanoparticles were determined to obtain *Commission International de l’Enlairage* (CIE L*a*b) color coordinates using a colorimeter. For this purpose, the most stable formulation obtained with a mass ratio of 5:1 was chosen, because no color differences were recorded among different ratios during storage (other ratios are not shown in the graph). The changes in a*-value (higher positive values indicate a redder color) at 4, 37 and 55 °C during storage for 21 days are shown in Figure 4. All the samples initially had a whitish color with negative a*-value, and changes in the a*-value of the control sample and PWP/DIM remained comparatively stable at a low temperature (4 °C) during storage. However, at a higher temperature of 37 or 55 °C the control sample (DIM only) became slightly red and the color intensity (a*-value) was significantly increased with increasing incubation time (*p* < 0.05) compared with encapsulated DIM, which remains stable during 37 or 55 °C treatment and very little changes were observed (Figure 4). The observed differences in the values describe the chemical degradation, and more light was absorbed by the native DIM at higher temperature compared to encapsulated DIM. Indole and carotenoids when exposed to heat and light undergo isomerization and photodegradation [51], and these results showed that DIM encapsulated in PWP nanoparticles was successfully protected from chemical degradation during heat treatment. This result is supported by studies showing that temperature can rise a*-values during storage, which suggests that emulsions became redder with the temperature rise. Temperature is the major factor causing isomerization, oligomerization, and oxidation of phytonutrients [45,52]. Heat treatments can cause color fading and chemical degradation in phytochemicals during storage [53]. Moreover, it was reported that DIM showed instability when exposed to heat and light, however, encapsulation might provide better protection [42].

**Figure 5:**
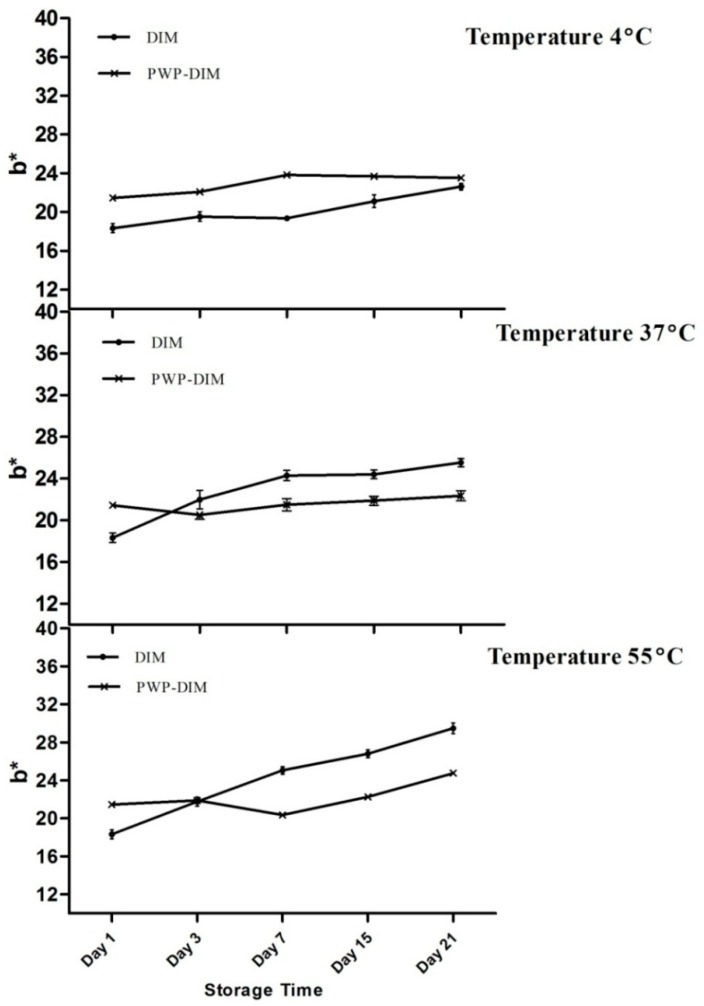
Effect of storage temperature (4, 37 and 55 °C) blue-yellow axis (b*-value) of DIM, and PWP/DIM with mass ratio (5:1) nanoparticles during storage. Values are mean ± SD (*n* = 3).

**Figure 6:**
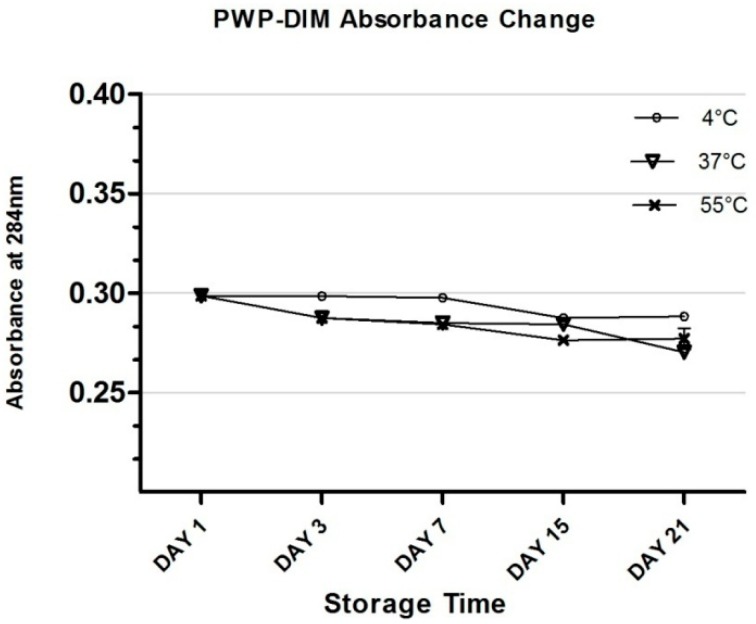
Effect of storage temperature (4, 37 and 55 °C) on the absorbance change of PWP/DIM with mass ratio (5:1) nanoparticles during storage. Values are mean ± SD (*n* = 3).

Figure 5 shows the b*-value (higher positive value means a more yellowish color) of all samples. Our results showed that very slight changes were obtained in the values at 4 °C for the control as well as in the encapsulated samples (Figure 5). However, a rapid and significant increase (*p* < 0.05) was found in the b*-value after storage for 21 days at 37and 55 °C in the control sample (DIM only), which indicated more yellowness compared with encapsulated DIM (Figure 5). Previous research found that there was a rapid increase in the b*-value of a lutein emulsion stored at 37 and 55 °C [53,54]. Overall, at low temperature (4 °C) the control sample and nanoparticles maintained a whitish color, but a slight change was observed with different incubation temperatures such as 37or 55 °C. Figure 6 shows the DIM content in polymerized whey protein (PWP) with a mass ratio PWP/DIM (5:1). DIM content was reduced in PWP by the decrease of UV absorbance for 21 days at the different temperature showed in (Figure 6).

DIM was significantly decreased in PWP after high temperature (37 or 55 °C) storage for 21 days, which could be due to loss of the limited amount of DIM which was not properly encapsulated. According to the previous literature, native DIM was found unstable under thermal conditions and degrades easily, however encapsulation provided better protection [50].

### 2.6. Changes in Particle Size and Zeta Potential of PWP–DIM Nanoparticles During Storage

**Figure 7:**
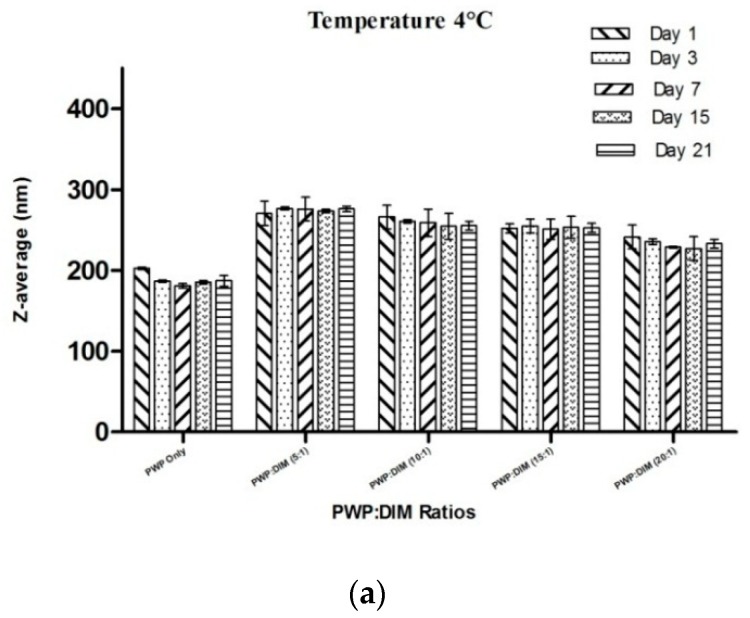

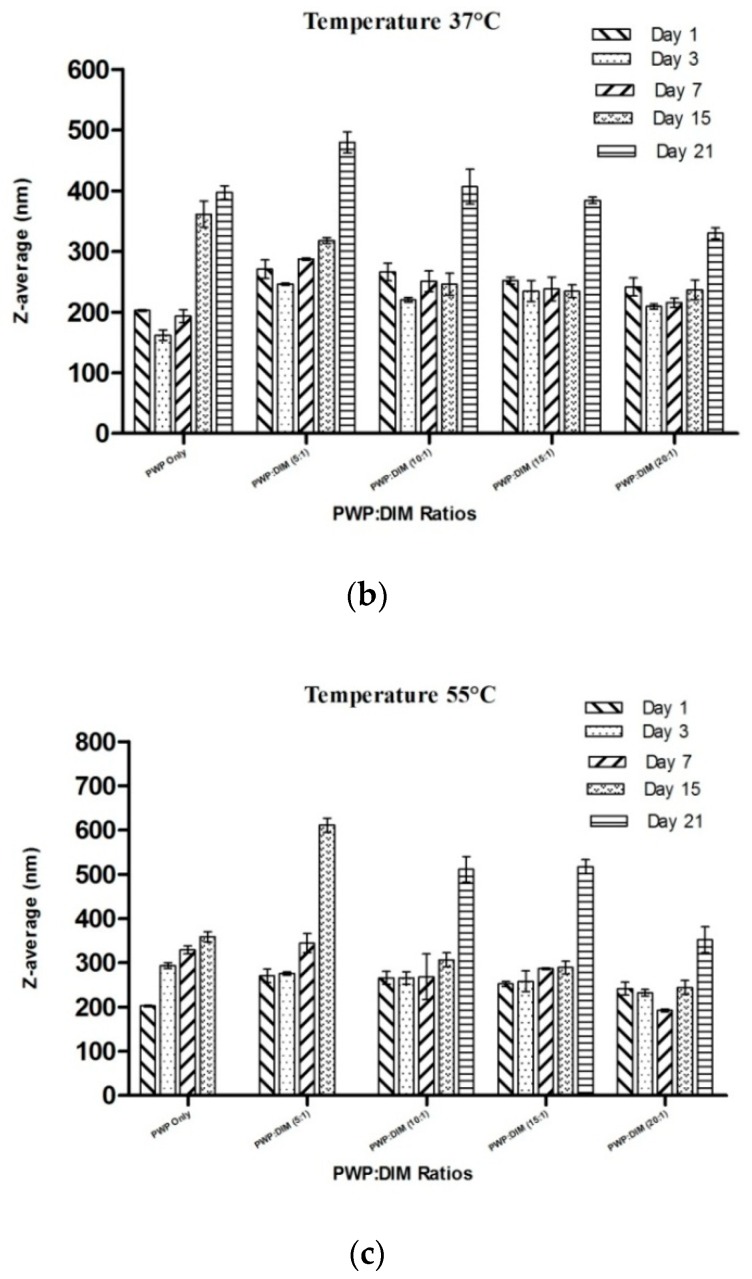
Effect of different storage temperature (**a**) 4 °C; (**b**) 37 °C; (**c**) 55 °C on the mean particle size of PWP/DIM nanoparticles with different ratios during storage. Values are mean ± SD (*n* = 3).

For most industrial applications, it is necessary that whey protein-based nanoparticles remain physically stable during storage. Temperature changes can affect the physical stability of the nanoparticles through various mechanisms. Heating can lead to aggregation and boost the frequency of particle collision sat a certain temperature [55]. Higher temperature can lead to conformational changes of any emulsifier such as whey protein by altering its ability to stabilize the solution against aggregation [52]. This impact was assessed by measuring the average particle size and zeta potential of PWP/DIM incubated for 21 days at 4, 37, and 55 °C (Figure 7). At 4 °C, the mean particle size of PWP samples was slightly decreased at first and remained stable over time. Mean particle size of the samples at different ratios of DIM in polymerized whey protein ranging from 5:1 to 20:1 were found to be significantly stable over the time at 4 °C (*p* < 0.05, Figure 7a). However, at higher temperatures of 37 or 55 °C, the overall changes in the mean size of PWP/DIM were comparatively small for all samples till day 15, but a significant stratification was observed at day 21 (Figure 7b,c). This significant increase showed that a higher temperature for a long duration can lead to droplet growth, especially the PWP/DIM (5:1 ratio), which showed a significant increase (*p* < 0.05) in the mean size over time, followed by the 10:1, 15:1 and 20:1 ratios. These results indicate an increase in the mean particle size during incubation. These findings are supported by different studies which revealed that the mean particle size of an emulsion was found stable at lower temperature, but the particle size increased at higher temperatures (25 and 37 °C) [56]. Other studies showed that β-lactoglobulin, a main component of whey protein, undergoes conformational changes such as surface denaturation during heat treatment, which exposes some of the non-polar groups in the protein structure [57]. Moreover, PWP only and PWP/DIM with mass ratio 5:1 formed a gel at 55 °C after 15days of incubation. 

Studies have revealed that the mean particle size of protein-stabilized nanoparticles and emulsions during storage at higher temperature could lead to increased particle size that could likely be attributed to aggregation and flocculation [57,58]. As a result, the number of disulfide bonds will increase with higher temperature, because when the temperature increases unfolding occurs and intermolecular disulfide bonds will form which may lead to flocculation. Another study on β-carotene solid lipid nanoparticles showed that particles was stable for 15 days compared to a significant increase (ranging from 354 to 2461 nm) in size for a sample heated up to 85 °C [53]. Studies suggest that heating can cause conformational changes such as surface denaturation in globular β-lactoglobulin with enhanced droplet aggregation through hydrophobic interaction [26,57]. Another reason may be the presence of ethanol in the system, which can also affect the emulsion in a concentration-dependent way [59].

**Figure 8:**
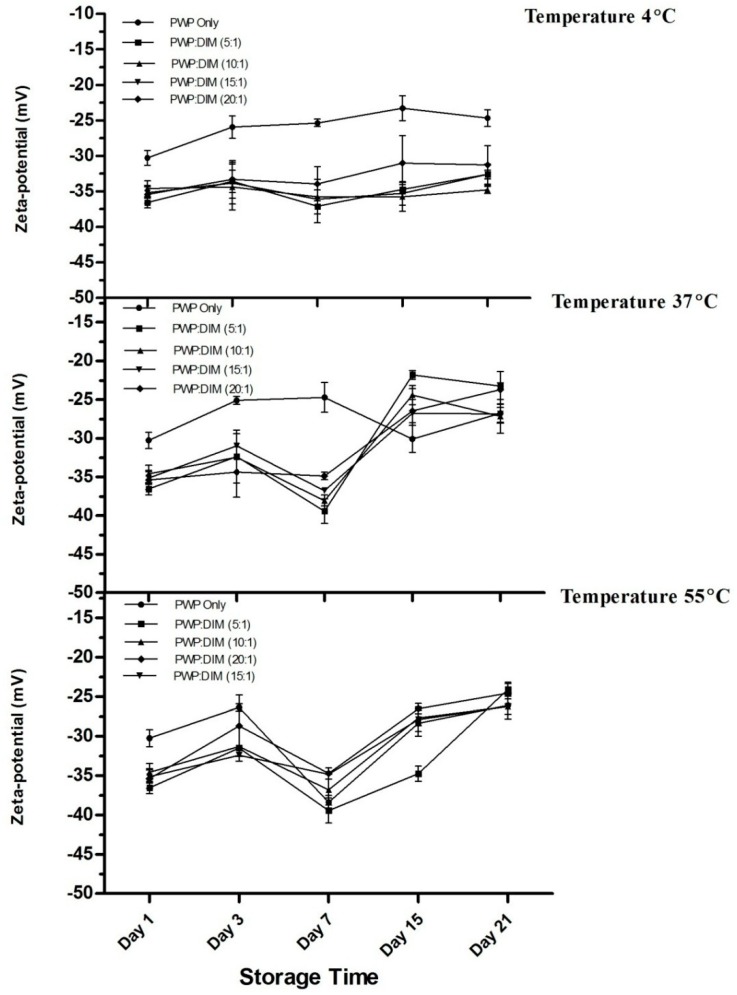
Effect of different storage temperatures (4, 37 and 55 °C) on the mean zeta potential of PWP/DIM nanoparticles with different ratios during storage. Values are mean ± SD (*n* = 3).

Zeta potential values for PWP/DIM a teach ratio and PWP only over 21 days at 4, 37, and 55 °C are shown in Figure 8. The zeta potentials of whey protein-based DIM nanoparticles were found to be highly negative (ranging from −36 to −31 mV) and stable during storage at 4 °C. However, incubation at higher temperature (37 or 55 °C) led to a reduction in zeta potential. The zeta potential values of DIM-PWP nanoparticles could led to a reduction during storage, which might be due to the binding of protein molecules on the surface and free molecules repelling each other [25,60]. Overall, all zeta potential data at different temperatures for all samples stored for 21 days were in the range from −25 to −30 mV, which is considered electrostatically stable, as reported previously [22]. Previous literature has revealed that storage of the samples can reduce zeta potential values at higher temperature (ranging from −16 mV to −15 mV), especially at 50 °C [34,35]. During storage duration above 37 °C, the zeta potential values of the nanoparticles were significantly decreased (*p* < 0.05). The decrease of zeta potential could be explained by the degradation/possible breakdown to secondary products of the carotenoids, which will produce highly reactive radicals due to oxidation [53].

## 3. Materials and Methods

### 3.1. Materials

3,3′-Diindolylmethane was purchased from LuotianXinpusheng Pharmaceutical Co., Ltd. (Hubei, China). Whey protein concentrate (WPC) was obtained from Hilmar (Turlock, CA, USA). Deionized water (filtered with a 0.22 μm filter) was purified using a Milli-Q deionization reversed osmosis system (Millipore Corp., Bedford, MA, USA).

### 3.2. Sample Preparation

PWP was prepared using the method explained by Wang et al. with modifications [26]. Whey protein solution (8%, *w*/*v*) was prepared with distilled water, and stirred at (1500 rpm) at ambient temperature for 2 h with a C-MAG HS7 magnetic stirrer (IKA, Staufen, Germany) and adjusted to pH 7.0 using NaOH solution (1 M). To ensure the complete hydration, solutions were refrigerated overnight at 4 °C. Whey protein solution (WP) was brought to 83 °C with constant stirring and kept for 20 min at this temperature, and equilibrated at room temperature. Diindolylmethane was prepared with pure ethanol as a stock solution, and was heated to 40 °C to form a clear solution under mild stirring. For preparation of whey protein based DIM nanoparticles (PWP/DIM), DIM solution was added to PWP solution to obtain a whey protein: DIM mass ratio ranging from 3:1 to 20:1, under mild stirring for 40 min. The mixtures were treated with ultrasound (VCX800, Vibra Cell, Sonics, Newtown, CT, USA) for 4 min at 30% amplitude with a 13 mm diameter probe (high-grade titanium alloy) placed in an ice bath to minimize heat gain. All samples were wrapped with aluminium foil to protect them from light.

### 3.3. Particle Size and Zeta Potential Measurement

Freshly prepared nanoparticle suspensions were used to measure the mean particle size and zeta potential using a Zeta sizer Nano ZS 90 (Malvern Instruments, Worcestershire, UK). For analysis, all the samples were diluted with deionized water to (1%) and analyzed at 25 °C. Each sample measurement was carried out in triplicates. The detection of particle size was conducted at a scattering angle of 173°.The zeta potential was calculated by the obtained electrophoretic mobility by the installed software based on the Henry equation. The polydispersity index (PDI) was also calculated automatically by the instrument to measure the particle size distribution. All the results were reported as mean ± SD.

### 3.4. Rheological Determinations

Rheological properties of samples were analyzed by a Hybrid Rheometer (TA Instruments, New Castle, DE, USA) connected with a cooling system (Thermo Cube, New York, NY, USA) equipped with a parallel plate (60-mm diameter; 1 mm thickness). Samples’ apparent viscosity was determined inthe shear rate range of 0.1 s^−1^ to 1000 s^−1^ at 25 °C with the gap width of 1000 μm.

### 3.5. Fourier Transform Infrared (FTIR) Spectra Analysis

The chemical structure of PWP only and whey protein based DIM nanoparticles were obtained by FTIR spectrophotometer (IR-Prestige 21, Shimadzu, Kyoto, Japan). All samples were freeze-dried and each sample (2.0 mg) was added to 200 mg potassium bromide and was ground to a fine powder for the preparation of the pellets. Spectra were obtained in the wavelength range of 400−4000 cm^‒1^ wave length.

### 3.6. Transmission Electron Microscopy (TEM) Analysis

All the samples were first diluted to 0.1% (*w*/*v*) concentration using ultrapure water. A droplet (~10 μL) was dropped onto a carbon-coated copper grid. Excess solution was eliminated by filter paper for 1 min and was air dried before imaging. All the samples were then stained with 2% (*w*/*v*) uranyl acetate solution for 2 min. The microstructures of the samples was examined by a Transmission Electron Microscope (H-7650, Hitachi High-Technologies, Tokyo, Japan) operated at a 100-kV acceleration voltage.

### 3.7. Storage Stability Analysis

The storage stability of all the samples was investigated. All the samples were stored in glass tubes with screw caps for 21 days at different temperatures (4, 37, and 55 °C), and data were taken at days 1, 3, 7, 15 and 21.

#### 3.7.1. Color and Absorbance Measurement

All samples were measured for changes in color and absorbance during storage at different temperatures. Color was assessed using a CM 2300d hand-held colorimeter (Konica Minolta, Osaka, Japan). Sample (10 mL) was poured onto a Petri dish and measurements were carried under dark conditions against a black ground. All readings were taken three times. The content of DIM was assessed from absorbance measurements (284 nm). All the samples were diluted to (0.1%) in DMSO (because it can dissolve both DIM and whey protein) to form a clear solution and then measured for absorbance using an UV-visible spectrophotometer (UV-2550, Shimadzu). The solution without DIM was used as a blank.

#### 3.7.2. Mean Particle Size and Zeta Potential Determination

All samples were determined for changes in mean particle size and zeta potential using a Zeta sizer Nano ZS 90 (Malvern Instruments) stored for 21 days.

### 3.8. Statistical Analysis

All experiments in this study were performed in triplicates. All the data were analyzed using a software program SPSS 20.0 (SPSS Inc. Chicago, IL, USA). The results are given as mean values and standard deviation. Comparison among the treatments of different groups was performed using an analysis of variance (ANOVA) with a post-hoc Tukey test. All tests were performed at a significance level of 0.05.

## 4. Conclusions

Whey protein nanoparticles were successfully developed to microencapsulate DIM. Whey protein based DIM nanoparticles were greater than whey protein alone in particle size and absolute zeta potential. The encapsulation of DIM was confirmed by FTIR spectra and TEM images analysis. Micro encapsulation of DIM by polymerized whey protein enhanced its storage stability at different temperatures for 15 days. The data of this study concluded that polymerized whey protein could be used to microencapsulate hydrophobic bioactive compounds, i.e., DIM for pharmaceutical and food applications.

## Figures and Tables

**Table 1:** Effects of different DIM to polymerized whey protein ratios on particle size, polydispersity index (PDI), zeta potential.

Sample	D_z_ (nm)	PDI	Zeta Potential (mV)
PWP Only	202.50 ± 1.56 ^a^	0.37 ± 0.0 ^a^	−30.26 ± 1.05 ^a^
PWP:DIM (5:1)	270.57 ± 15.28 ^b^	0.61 ± 0.05 ^b^	−36.56 ± 0.73 ^b^
PWP:DIM (10:1)	265.97 ± 14.51 ^b^	0.42 ± 0.01 ^ca^	−34.60 ± 1.12 ^b^
PWP:DIM (15:1)	252.00 ± 5.63 ^b^	0.44 ± 0.01 ^c^	−35.13 ± 0.83 ^b^
PWP:DIM (20:1)	241.33 ± 14.82 ^b^	0.44 ± 0.05 ^c^	−35.40 ± 1.24 ^b^

Note. Values with different superscript letters within a column denote a significant different among different DIM ratios in PWP nanoparticles at (*p* < 0.05).

**Table 2:** Infinite-shear-rate viscosity, consistency index and flow index were derived from the fitting results of flow curves with Sisko Model.

Samples	*η*_∞0_ (mPa∙s) Infinite-Shear-Rate Viscosity	*k*_0_ Consistency Index	*n*_0_ Flow Index
PWP Only	5.205 ± 0.014 ^a^	386.701 ± 10.141 ^ab^	0.016 ± 0.006 ^a^
PWP:DIM (5:1)	6.344 ± 0.011 ^b^	390.108 ± 6.4156 ^a^	0.046 ± 0.004 ^b^
PWP:DIM (10:1)	6.470 ± 0.023 ^c^	392.158 ± 16.327 ^ab^	0.010 ± 0.010 ^c^
PWP:DIM (15:1)	5.656 ± 0.011 ^d^	441.536 ± 8.677 ^c^	−0.004± 0.005 ^d^
PWP:DIM (20:1)	5.340 ± 0.034 ^e^	537.473 ± 34.462 ^d^	−0.049 ± 0.016 ^e^

Note. Values with different superscript letters within a column denote a significant different among different DIM ratios in PWP nanoparticles at (*p* < 0.05).
